# Evaluation of the RUBY modular QA phantom for planar and non‐coplanar VMAT and stereotactic radiations

**DOI:** 10.1002/acm2.13006

**Published:** 2020-08-14

**Authors:** Daniela Poppinga, Jana Kretschmer, Leonie Brodbek, Jutta Meyners, Bjoern Poppe, Hui Khee Looe

**Affiliations:** ^1^ PTW Freiburg Freiburg Germany; ^2^ University Clinic for Medical Radiation Physics Medical Campus Pius Hospital Carl von Ossietzky University Oldenburg Germany; ^3^ Department of Radiation Oncology University Medical Center Groningen University of Groningen Groningen Netherlands; ^4^ Radiotherapy Department Imland Hospital Rendsburg Germany

**Keywords:** couch check, End‐to‐End testing, SRS QA, system QA

## Abstract

**Purpose:**

This study evaluates the clinical use of the RUBY modular QA phantom for linac QA to validate the integrity of IGRT workflows including the congruence of machine isocenter, imaging isocenter, and room lasers. The results have been benchmarked against those obtained with widely used systems. Additionally, the RUBY phantom has been implemented to perform system QA (End‐to‐End testing) from imaging to radiation for IGRT‐based VMAT and stereotactic radiations at an Elekta Synergy linac.

**Material and Methods:**

The daily check of IGRT workflow was performed using the RUBY phantom, the Penta‐Guide, and the STEEV phantom. Furthermore, Winston–Lutz tests was carried out with the RUBY phantom and a ball‐bearing phantom to determine the offsets and the diameters of the isospheres of gantry, collimator, and couch rotations, with respect to the room lasers and kV‐imaging isocenter. System QA was performed with the RUBY phantom and STEEV phantom for eight VMAT treatment plans. Additionally, the visibility of the embedded objects within these phantoms in the images and the results of CT and MR image fusions were evaluated.

**Results:**

All systems used for daily QA of IGRT workflows show comparable results. Calculated shifts based on CBCT imaging agree within 1 mm to the expected values. The results of the Winston–Lutz test based on kV imaging (2D planar and CBCT) or room lasers are consistent regardless of the system tested. The point dose values in the RUBY phantom agree to the expected values calculated using algorithms in Masterplan and Monte Carlo engine in Monaco within 3% of the clinical acceptance criteria.

**Conclusion:**

All the systems evaluated in this study yielded comparable results in terms of linac QA and system QA procedures. A system QA protocol has been derived using the RUBY phantom to check the IGRT‐based VMAT and stereotactic radiations workflow at an Elekta Synergy linac.

## 1 Introduction

The process of radiation therapy from patient imaging for the treatment planning until the radiation at the linear accelerator (linac) requires seamless integration of multiple system components along the chain. Any errors that occur within any system components could affect the quality of the treatment plan delivered and hence the patient's clinical outcome. Therefore, stringent tests are designed and carried out either at regular intervals or on a patient‐to‐patient basis aiming at detecting these errors before the treatment begins and during the course of the treatment. There exist several guidelines, which recommend quality assurance (QA) protocols for specific system components, like the TG 142^1^ for linac, the TG 179^2^ for CT‐based IGRT workflow, the AAPM practical guideline 5.a^3^ for treatment planning system, and the TG 218^4^ for the patient‐specific plan verification.

In view of emerging technologies in radiation therapy, the complexity of the radiation therapy process has increased, partly due to the integration of additional system components into the treatment chain like onboard cone‐beam CT (CBCT) imaging systems or optical body scanning systems as used for surface‐guided radiation therapy (SGRT). Image‐guided radiation therapy (IGRT) relies on the daily image acquisitions to identity errors in patient immobilization and positioning. The results from the image guidance routine are derived based on either an automated or a manual registration workflow between the daily and the reference images. The execution of couch corrections resulted from this procedure involves the communication between different system components, such as the imaging software, the Record and Verify (R&V) system, and the couch control system. Adaptive radiation therapy also relies heavily on the use of onboard imaging, but not only for the verification of patient positioning, but also for plan adaption based on daily anatomical changes assessed from these images. The QA of this workflow involving multicomponents is essential for correct patient treatment and in case of remote controlled couch system, a daily‐based QA workflow is also recommended. Thereby, it is essential to implement check procedures to ensure the congruence of the isocenters of the imaging systems and the linac. This can be realized for example by performing the procedure introduced by Lutz et al^5^ as recommended in TG 179.

However, even if QA tasks within each system component are passed, the correctness of the complete radiation therapy process is still not guaranteed as the outcome necessitates the faultless interplay between different system components due to their underlying dependencies. Therefore, a unified system QA, also referred to as End‐to‐End testing, that covers the entire process from the beginning to the end is desired to identify these system dependencies and any flaws within the whole radiation therapy process. The system QA should also ensure the data integrity when information is passed between various system components. During the execution of the system QA, the tasks should be carried out by the same clinical team members, who are performing these tasks clinically, so that possible faults caused by user's interactions with these system components can be pinpointed.

No universal protocols for system QA are available due to the diversity of the clinical systems in use and institutional specific workflows. The system QA should be designed to reflect every aspect and system component along the chain of a radiation therapy process as clinically realistic as possible. This execution of the system QA can be considered as a dry‐run of the whole process, only without the patient. This suggests the need to use a patient surrogate, typical a phantom or ideally, an anthropomorphic phantom.

In this work, the clinical use of a new modular phantom (RUBY, PTW Freiburg, Germany) has been evaluated as a universal system QA phantom for VMAT irradiations as well as IGRT‐specific QA in terms of geometry accuracy checks. The performance of the new phantom is compared to an established head anthropomorphic phantom^6^ (STEEV, CIRS, Norfolk, USA) used for system QA of intracranial stereotactic radiation, a widely used IGRT QA phantom^7^ (Penta‐Guide, Modus Medical Devices Inc, London, Canada); and a ball‐bearing system (Elekta, Stockholm, Sweden).

## 2 Materials and Methods

### 2.A Linear accelerator and treatment planning systems

Measurements were performed at an Elekta Synergy linac with Agility MLC (Elekta, Stockholm, Sweden). The machine is equipped with both MV (iViewGT, Elekta, Stockholm, Sweden) and kV (XVI, Elekta, Stockholm, Sweden) onboard imaging systems. Three‐dimensional (3D) CT scans were performed for all the used phantoms separately for each task‐specific modular insert (see below) at a Siemens Sensation 64 CT (Siemens Healthineers, Erlangen, Germany) equipped with Elekta treatment table iBEAM evo. All scans were reconstructed in 1 mm slice thickness. Treatment table was included in the external structure. Dose calculations were performed using a voxel size of 2 mm × 2 mm × 1 mm (slice thickness). Additionally, magnetic resonance imaging (MRI) was performed for the RUBY phantom with the System QA insert as well as for the STEEV phantom with the MRI insert at a Siemens MAGNETOM Verio with 3 T (Siemens Healthineers, Erlangen Germany) using T1 and T2 sequences. The R&V System MOSAIQ (Elekta, Stockholm, Sweden) was used with the Synergistic (Elekta, Stockholm, Sweden) workflow implemented.

Eight VMAT plans with 6 MV flattened beam were created using Oncentra Masterplan (Elekta, Stockholm, Sweden) (see Table 1), which were optimized and calculated using the collapsed cone algorithm. The studied treatment plans include six coplanar radiations with dose per fraction between 1.8 Gy and 6 Gy and two non‐coplanar radiations consisting of four different couch angles with dose per fraction of 6 Gy. The target volume sizes range between 4.8 cm^3^ and 486 cm^3^.

**Table 1:** Overview of the treatment plans studied. Six coplanar treatment plans with dose per fraction between 1.8 Gy and 6 Gy. Two non‐coplanar treatment plans with dose per fraction of 6 Gy. Target volume sizes are between 4.8 and 486 cm^3^.

Plan entity	Fraction dose [Gy]	Monitor units	Target volume [cm^3^]	Arc length [degree]/ Couch Rotation [degree]		Fraction dose [Gy]	Monitor units	Target volume [cm^3^]	Arc length [degree]/Couch Rotation [degree]
Prostate	1.8	382.8	239.8	360/0	Plan entity	6	427.8	16.2	360/0
Glioblastoma	2	277.0	486.2	360/0	Brain metastasis	6	726.8	14.2	360/0
Brain metastasis	6	783.7	15.8	360/0	Brain metastasis	6	319.7	4.8	360/0
							206.5		180/45
							157.4		180/315
							176.2		180/270
Brain metastasis	6	809.0	4.8	360/0	Brain metastasis	6	403.5	16.2	360/0
							127.1		180/45
							147.6		180/315
							145.5		180/270

### 2.B Phantoms

#### 2.B.1 RUBY phantom

The RUBY phantom (PTW Freiburg, Germany) is a modular polystyrene phantom system consisting of a base body and four modular inserts. The phantom's surface has three sets of markers: the black lines indicate the center of the phantom base; the other two sets of lines are used to misalign the phantom in a defined manner (gray lines for translational misalignment; red lines for a combination of translational and rotational misalignment). The rotational misalignment of the phantom can be achieved with a tilted base plate. The functionality of each insert is described in the following.

The Linac QA insert consists of four bone equivalent cylinders distributed in all spatial directions to provide sufficient data for CBCT image registration. A ceramic ball of 8 mm in diameter at the center of the insert allows the performance of a Winston–Lutz test.

The Patient QA insert is a homogeneous polystyrene insert in which a detector can be inserted for point dose measurement at the center of the phantom. The insert is compatible with different detector types. This insert was not evaluated in this work separately as point dose measurement as treatment plan verification is performed as part of the System QA.

The System QA insert contains three tubes (1 cm, 1.5 cm, and 2.5 cm diameter) filled with a MRI visible liquid and three additional tubes with lung equivalent material (3 cm diameter), bone equivalent material (1.8 cm diameter), and brain equivalent material (2.2 cm diameter). Like the Patient QA insert, different detectors can be inserted allowing point dose measurements at the center of the phantom. All structures are positioned around the detector, and in addition, the structures are interrupted at different positions to enable 3D image registration.

#### 2.B.2 Phantom Patient for Stereotactic End‐to‐End Verification (STEEV)

STEEV phantom (CIRS, Norfolk, USA) is an anthropomorphic head phantom with realistic anatomical details for the End‐to‐End testing of stereotactic radiations. The phantom can be used with multiple inserts that allow each step from CT, MR, or PET imaging to plan irradiation to be checked. In this work, three inserts were used: the geometric machine QA insert (part number 038‐08), the target insert (038‐03) for the microDiamond detector (type 60019, PTW Freiburg, Germany), and the MRI insert (part number 038‐11). The machine QA insert consists of two different spheres with high‐z material visible in CT. One is in the true isocenter, which is designated by the markers on the phantom's surface. The second sphere is located at defined translational shifts apart from the first sphere. The distance between the spheres is 15 mm, 25 mm, and 20 mm in lateral, longitudinal, and vertical direction, respectively. The target insert consists of a 30‐mm sphere target. The detector‐specific insert allows point dose measurement at the center of the target. The MRI insert consists of a water‐filled sphere of the same size as the target. The surrounding volume within the insert is also filled with water.

#### 2.B.3 Penta‐Guide

The Quasar Penta‐Guide Phantom (Modus Medical Devices Inc., London, Canada) is designed for the daily testing of IGRT workflow, especially for the execution of automatic couch corrections. It is a widely used phantom for this purpose by Elekta linac users. The quadratic phantom contains five low‐density rings and hollow spheres that serve the purpose of image registration for different modalities such as onboard kV and MV planar and CBCT image acquisitions. Dose measurement is not possible with this phantom as it only serves the purpose of identifying isocenter's misalignment.

#### 2.B.4 Ball‐bearing phantom

The ball‐bearing phantom (Elekta, Stockholm, Sweden) consists of a ball bearing with diameter of 8 mm made of steel that is embedded in an acrylic wand. The wand is mounted on a three‐dimensional positioning stage that can be adjusted using the built‐in micrometers, with which the ball bearing can be positioned at the imaging isocenter with the help of planar images or CBCT. Subsequently, the room lasers can be tuned to align with the markers on the phantom.

### 2.C Detectors

The PinPoint 3D (type 31022) ionization chamber and microDiamond (type 60019) detector (both PTW Freiburg, Germany) were used for single point treatment plan verification in conjunction with the RUBY and STEEV phantoms. The microDiamond detector is cross‐calibrated against a reference class Semiflex 3D (type 31021) ionization chamber (PTW Freiburg, Germany) using the RUBY phantom with homogenous insert. The PinPoint 3D chamber was used without cross‐calibration. If not otherwise stated, measurements were performed according to the TRS 483. Nevertheless, due to lack of composite field correction factors, no field size‐dependent correction factors were applied to the measurements. The consequences are discussed in terms of measurement uncertainty in the following sections.

The difference between measurement and TPS calculation was determined using the following formula:

(1) $$Diff\left(\%\right)=\left(1‐\text{TPS}\;/\text{Measurement}\right)\cdot 100$$

where a positive difference value indicates that the measurement value is larger than the TPS calculation and vice versa.

### 2.D Linac quality assurance

The purposes of linac quality assurance using the phantoms evaluated in this work are to check (i) the integrity of IGRT and couch correction workflow; and (ii) the congruence of the linac rotational and imaging isocenters.

**Fig. 1:**
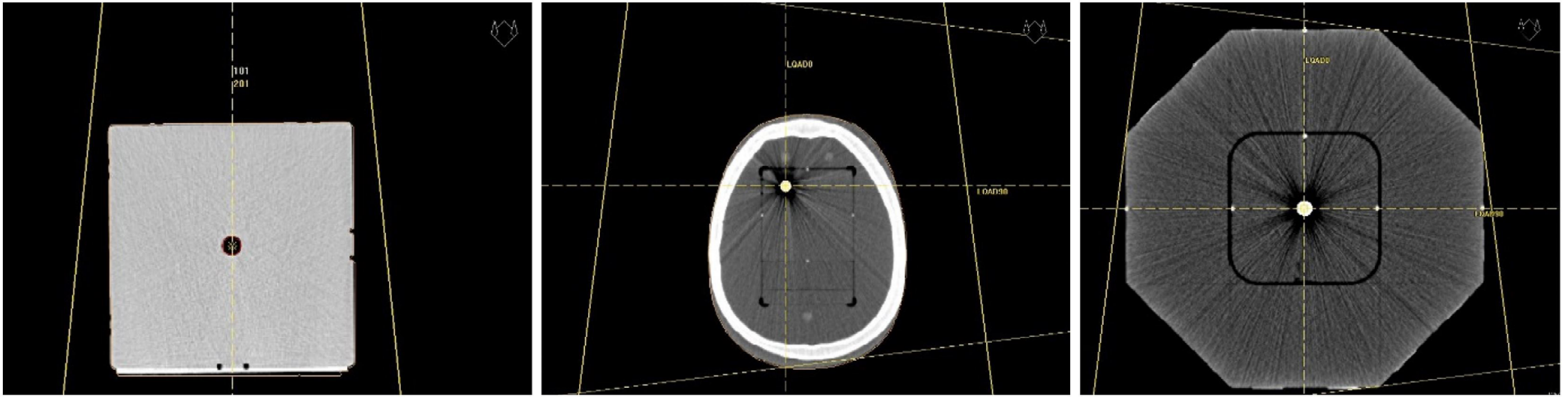
Left: Penta‐Guide phantom with defined isocenter position according to the air‐filled central sphere. Middle: STEEV phantom with defined isocenter position at the center of to the off‐axis high‐Z sphere. Right: RUBY phantom with defined isocenter position according to the CT markers.

In order to set up an IGRT — check of automated couch correction Planning CTs of Penta‐Guide, STEEV with machine QA insert and RUBY with Linac QA insert were imported to the Oncentra Masterplan TPS. In case of the Penta‐Guide phantom, the isocenter was set at the center of the central air‐filled sphere. In case of STEEV, the isocenter was set at the center of the off‐axis high‐density sphere, and in case of RUBY, the isocenter was set according to the six CT markers as shown in Fig. 1 for all phantoms. For each phantom, the whole phantom body was included in the external contour and orthogonal setup fields of 20 cm × 20 cm size and the corresponding DRRs were created. Each plan of each phantom alongside with the generated DRRs, the DICOM‐RT structure set, and the planning CT were exported to the MOSAIQ V&R system.

In the treatment room, each phantom was positioned on the treatment couch using the room lasers. Penta‐Guide and RUBY phantoms were positioned according to the false isocenter markings (gray lines of the RUBY phantom). The STEEV phantom was positioned according to the markings of the phantoms central sphere.

For each phantom, a set of planar orthogonal setup images was acquired using 6 MV beam and 2 monitor units. The pair of orthogonal images was manually registered to the corresponding DRRs using the stereoscopic matching function of MOSAIQ. In addition, CBCT images were acquired using the “Fast Head and Neck” preset in case of STEEV and Penta‐Guide and using the “Pelvis” preset in case of RUBY. Automatic image registrations were performed with the clinically used “Bone (T + R)” preset for the STEEV and RUBY phantoms. In case of the Penta‐Guide phantom, the “Grey values (T + R)” preset had to be used, since no bone structures are embedded in the phantom.

#### 2.D.1 Geometry accuracy

Winston–Lutz tests were performed using the RUBY phantom with the Linac QA insert as well as the ball‐bearing phantom. The RUBY phantom and the ball‐bearing phantom were positioned in two different ways. In the first method, both phantoms were positioned according to the room lasers. The second positioning approach was based on the use of kV planar images. In case of the kV planar images, the phantom was positioned in a way that the high‐density sphere was aligned to the projected imaging isocenter in the kV image. In addition, the RUBY phantom was also positioned by CBCT imaging with the associated automated image registration and couch corrections.

For each test, a series of planar images were acquired using a 3 cm × 3 cm field size at 6 MV with 10 monitor units by rotating the gantry and collimator from 0 to 360 degree in 30 degree step; and the couch from 90 to 270 degree in 15 degree step. For both phantoms, each image series was analyzed with the IsoCheckEPID software (PTW Freiburg, Germany) using the “minimum sphere” option.

### 2.E System (End‐to‐End) QA

MR imaging was performed for the RUBY phantom with the System QA insert as well as for the STEEV phantom with the MRI insert. Additionally, planning CT was acquired for both phantoms (RUBY phantom with System QA insert and STEEV with target insert). The acquired images were stored into the institution's PACS system, and from there, the images were exported and subsequently imported into the TPS by a dosimetrist. The MR images were registered to the planning CT using the mutual information algorithm with standard settings but by limiting the ROI to the regions, where the MR visible structures are located. Only external contours were delineated in the planning CT that encompass the complete phantom bodies.

In the next step, the patient treatment plans in Table 1 were imported into the planning CT of the RUBY and STEEV phantoms in Masterplan and Monaco, on which the dose distributions have been calculated using pencil beam and collapsed cone algorithms in Masterplan with inhomogenity correction turned on and the Monte Carlo engine in Monaco. Although only collapsed cone algorithm and Monte Carlo engine are used clinically, the results from pencil beam are provided here for comparison purposes.

The plans’ isocenters were set according to the CT markers and the dose distributions were computed. The plans with a pair of orthogonal setup fields with the corresponding DRRs, structure set, and planning CT were exported into MOSAIQ. The plans were then checked and approved by a physicist. At the linac, the further preparations required for the IGRT workflow in MOSAIQ and XVI were carried out by a radiation therapist. These steps include, for example, the transfer of data from MOSAIQ to XVI and the definitions of ROI and registration protocol in XVI.

Subsequently, the RUBY phantom was positioned using the room lasers according to the false markings on the phantom surface (without rotational errors). CBCT was acquired and the resulting couch corrections in terms of translational shifts were sent to the linac via MOSAIQ. Since there are no predefined false markings on the STEEV phantom, it was positioned according to the markers directly. The same IGRT workflow was executed to correct for the residual positioning errors. After the couch corrections, the treatment plans were irradiated and measured with the microDiamond detector. The PinPoint 3D chamber was used additionally in combination with the RUBY phantom. The measured point dose values were then compared to the TPS‐calculated values.

## 3 Results

### 3.A Linac quality assurance

#### 3.A.1 IGRT — check of automated couch correction

**Fig. 2:**
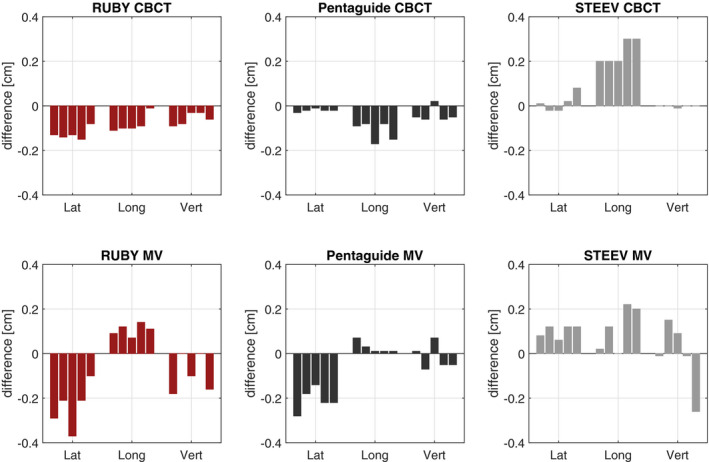
Difference between defined misalignment and detected misalignment according to Table 2 for Penta‐Guide, STEEV, and RUBY phantoms using CBCT and MV planar image acquisition.

The mean detected misalignment values for five independent couch correction checks using CBCT and MV planar images are compared to the expected values in Table 2 for the three phantoms investigated and the difference between expected and detected shift is shown in Fig. 2. For CBCT imaging, the calculated shifts in lateral and vertical directions agree within 1.6 mm to the expected values. For the longitudinal direction, the deviation is up to 3 mm for all phantoms. The standard deviation for CBCT imaging is less than 0.5 mm. In case of MV planar imaging, the agreement between the calculated shifts and expected values is up to 3.7 mm. It is noteworthy that the image registration of the MV planar images was performed manually, whereas the image registration of the CBCT images was automated based on the implemented algorithm in the software.

**Table 2:** Detected misalignment values (mean value and standard deviation for n = 5) for Penta‐Guide, STEEV, and RUBY phantoms using CBCT and MV planar image acquisition.

		**Penta‐Guide**		**STEEV**		**RUBY**	
		Defined misalignment [cm]	Detected misalignment [cm]	Defined misalignment [cm]	Detected misalignment [cm]	Defined misalignment [cm]	Detected misalignment [cm]
**CBCT**	Lateral	−1.00	−0.98 (0.01)	1.50	1.51 (0.04)	2.50	2.37 (0.03)
	Longitudinal	1.40	1.29 (0.04)	2.50	2.74 (0.05)	1.40	1.32 (0.04)
	Vertical	1.20	1.16 (0.03)	2.00	2.00 (0.00)	1.80	1.74 (0.03)
**MV planar**	Lateral	−1.00	0.80 (0.06)	1.50	1.60 (0.03)	2.50	2.26 (0.10)
	Longitudinal	1.40	1.43 (0.03)	2.50	2.61 (0.10)	1.40	1.51 (0.03)
	Vertical	1.20	1.19 (0.06)	2.00	1.99 (0.16)	1.80	1.71 (0.09)

#### 3.A.2 Geometry accuracy

The calculated diameters of the isospheres resulted from the Winston–Lutz tests for gantry, collimator, and table rotations performed with RUBY phantom and ball‐bearing phantom are presented in Table 3 for the three methods of positioning.

**Table 3:** Calculated isosphere diameters and offset positions for all performed Winston–Lutz test series.

	RUBY (Laser)	Ball bearing (Laser)	RUBY (planar kV)	Ball bearing (planar kV)	RUBY (CBCT)
	Diameter [mm]	Diameter [mm]	Diameter [mm]	Diameter [mm]	Diameter [mm]
Gantry	1.3	1.66	1.29	1.29	1.35
Collimator	0.39	0.56	0.59	0.49	0.53
Table	2.23	2.09	2.74	3.39	2.74

**Fig. 3:**
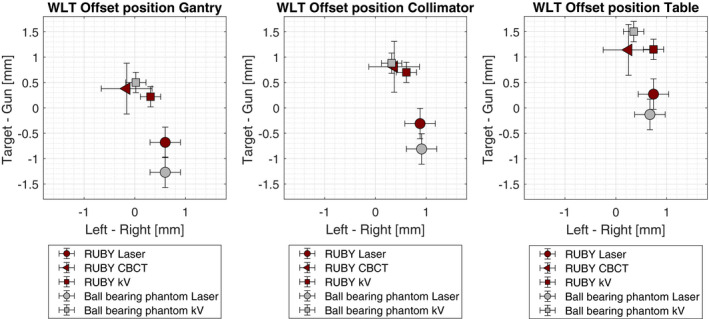
Calculated offset values in lateral (Left–Right) and longitudinal (Target–Gun) directions for both systems (RUBY phantom and ball‐bearing phantom) and three positioning methods (room lasers, planar kV imaging, CBCT imaging).

**Fig. 4:**
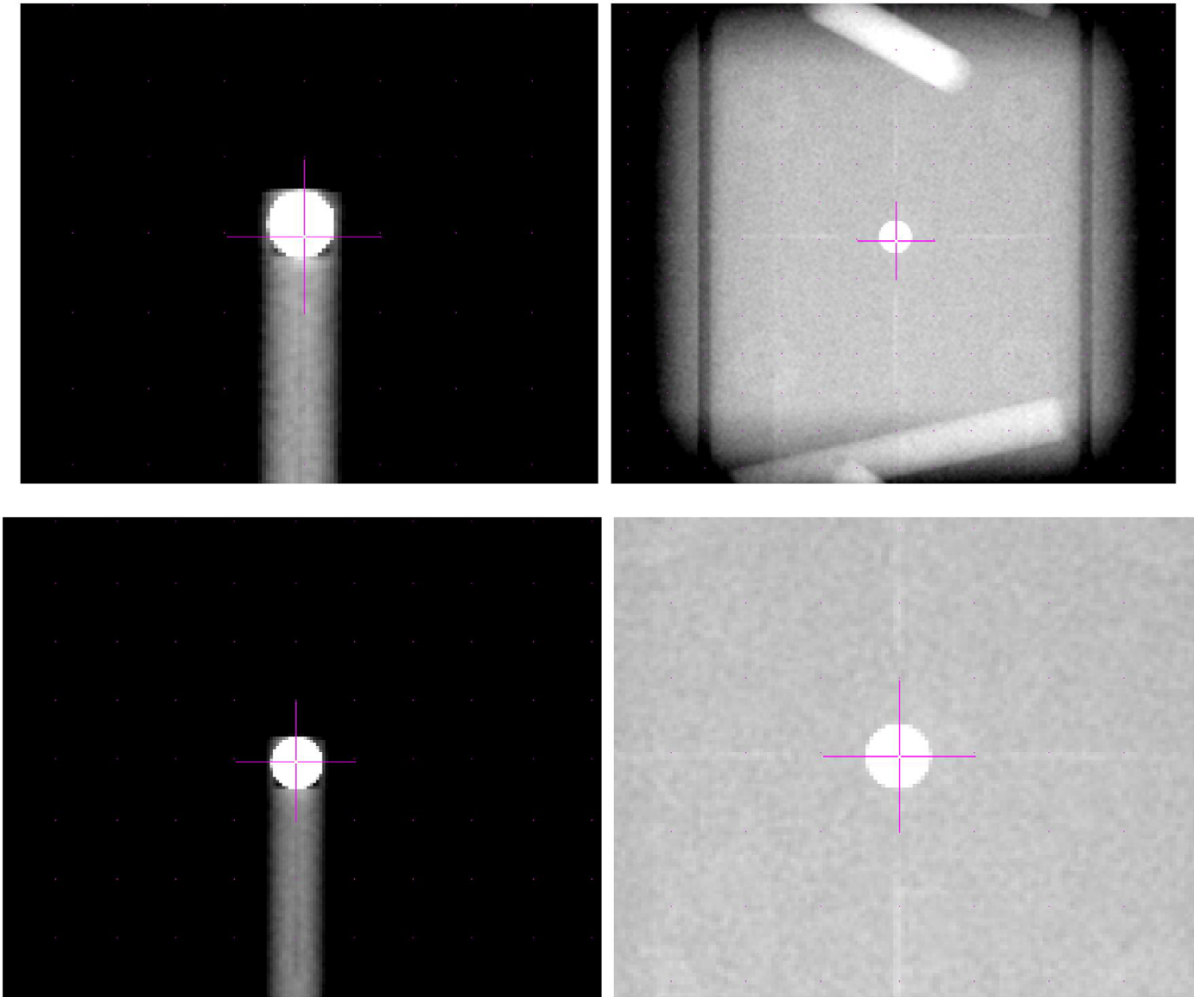
Planar kV images with the projected kV isocenter position of ball‐bearing phantom (left) and RUBY phantom (right). Upper row shows the positioning according to the room lasers. Lower row shows the positioning according to the projected kV isocenter position.

The calculated offset positions are plotted in Fig. 3. The uncertainty of the positioning method is different in each case. According to the results in Table 2, a value of 0.5 mm was assumed for the CBCT uncertainty. An uncertainty of 0.3 mm was assumed for the positioning using the room laser system and an uncertainty of 0.2 mm was taken for the positioning using the kV planar images as shown in Fig. 4. The results obtained based on kV imaging (2D planar and CBCT) or room lasers are consistent regardless of the system used. Nevertheless, the results show distinct differences between these two groups of data indicating a shift between the room lasers and the kV isocenter in the longitudinal direction of approximately 1.5 mm. This has been illustrated in Fig. 4 that shows the AP planar kV images with the RUBY phantom and the ball‐bearing phantom positioned according to the room lasers (upper row) and after the alignment of these images to the projected kV isocenter (lower row). There is a visible shift in longitudinal direction of approximately 1.5 mm before and after the alignment. This result is consistent with the results according to Table 2, where all three phantoms showed a deviation in the longitudinal direction.

### 3.B System (End‐to‐End) QA

**Fig. 5:**
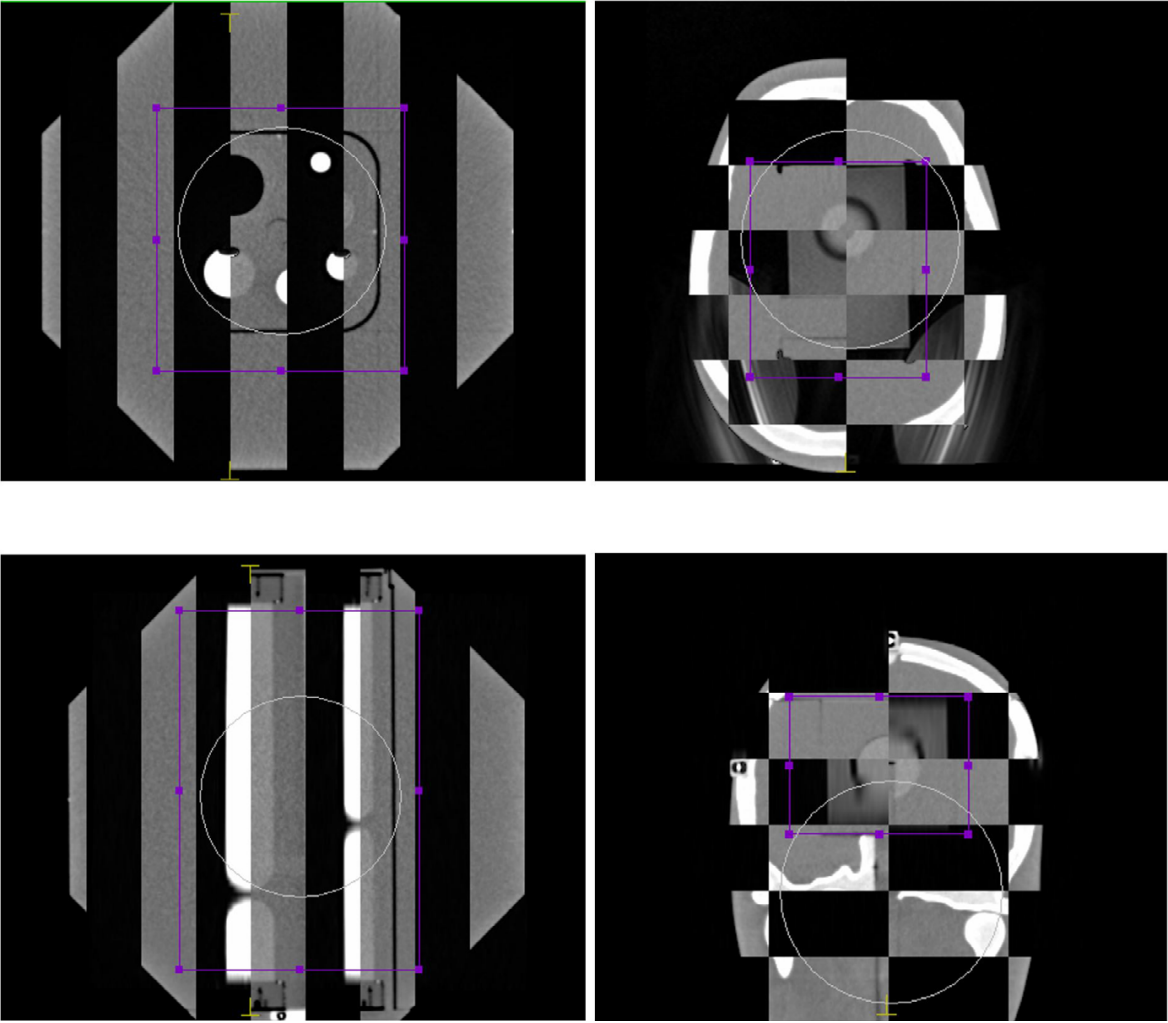
MRI CT image registration. Left column: RUBY with System QA insert Right column: STEEV phantom with MRI insert.

Figure 5 shows the registration results of the MR images of the RUBY with the System QA insert and the STEEV phantom with the MRI insert in Monaco TPS. The quality of the registration was assessed visually by comparing the geometry and positions of the MR visible structures in both datasets after the registrations. For both phantoms, the registration in Monaco yielded clinically satisfactory results. In case of RUBY, the registration can be evaluated based on the spatial information (translational and rotational correlations) of the MRI visible cylindrical structures. The goodness of the registration between CT and MRI was assessed by evaluating the coincidence of the circular cross sections of the rods with different radiuses in the transversal layers and of the discontinuities built in to the cylindrical structures in the coronal orientation. No visible discrepancy between CT and MRI could be asserted in both cases as demonstrated in Fig. 5. In case of STEEV, the image registration was evaluated based on the MRI visible spherical structure, where also no visible spatial misalignment was found as well. However, the registrations with both phantoms did not yield satisfactory results with the default settings in Masterplan.

**Fig. 6:**
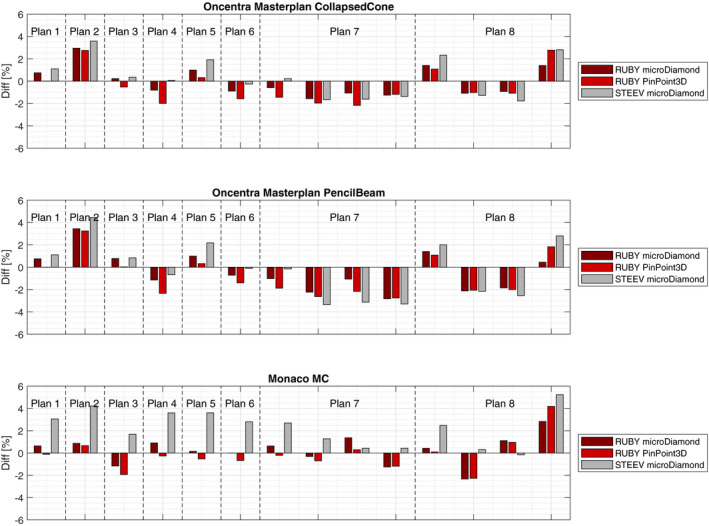
Differences between measurements in RUBY phantom with System QA insert or STEEV phantom with microDiamond insert and TPS calculations using collapsed cone (upper panel), pencil beam (middle panel), and Monte Carlo (lower panel) algorithms. Measurements were performed with microDiamond and PinPoint 3D ionization chamber in case of RUBY and microDiamond only in case of STEEV. Before the measurements, the IGRT workflow was performed using CBCT imaging.

The results from the point dose measurements performed after both the RUBY and STEEV phantoms were positioned following the CBCT‐based IGRT workflow are shown in Fig. 6. For the non‐coplanar treatment plans (plan 7 and 8), the results are presented individually for each arc. The measured dose values in RUBY phantom using both the microDiamond detector and PinPoint 3D chamber agree to the expected values calculated using the collapsed cone and pencil beam algorithm in Masterplan within 3%, except for plan 2, where a slightly higher deviation (3.4%) was observed when compared to the pencil beam calculations. The comparison of the microDiamond measurements with the STEEV phantom to the calculations of Masterplan is comparable to the results obtained with the RUBY phantom.

Measurements with the RUBY phantom also agree to the Monaco calculations using the Monte Carlo dose engine. All measured values agree within 3% to the calculated dose values except for one arc in plan 8. As shown in Fig. 6 (lower panel), larger deviations of up to 5.2% (plan 8, arc 4) are observed for the STEEV phantom when compared to Monaco calculations. It is noteworthy that, despite the differences in detector type, both the microDiamond detector and PinPoint 3D chamber yielded comparable results, with deviations less than 1.4% for all the investigated treatment plans.

## 4 Discussion

**Fig. 7:**
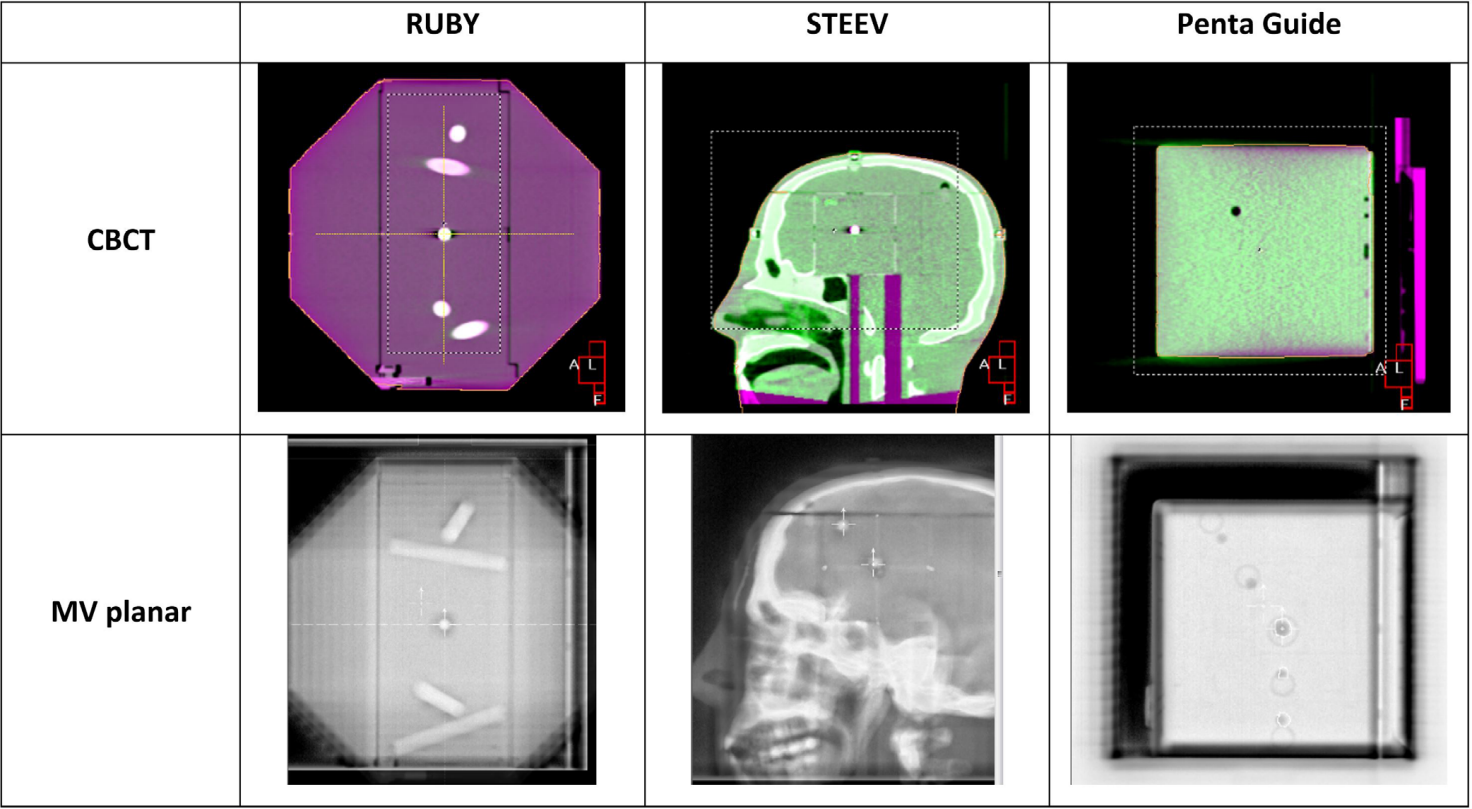
Example images of CBCT and MV planar acquisitions using the three phantoms investigated for the routine QA of the IGRT workflow.

The results from this study demonstrated that all three systems, RUBY phantom with the Linac QA insert, STEEV phantom with the machine QA insert and Penta‐Guide, are suitable tools for the routine daily QA of the clinical IGRT workflow that involves manual or automatic image registrations and automatic couch corrections. Nevertheless, the Penta‐Guide and the RUBY phantoms with their more compact form factor allow for easier handling and positioning for use as a daily check device. The STEEV phantom with realistic anatomical structures provides more contrast details that could ease the image registration process, especially during the manual registration of planar images. The low‐density cavities within the Penta‐Guide phantom resulted in lower contrast in the MV planar images. Furthermore, CBCT registration could only be performed using the “Grey values (T)” or “Grey values (T + R)” presets and not the clinical “Bone T + R” preset due to the absence of high density bone‐like structures. In contrast, the bone cylinders in the RUBY Phantom allow clearer visibility within the MV planar images and the use of the clinical “Bone T + R” preset during CBCT imaging. The differences in the visibility of the embedded objects used for the image registrations are shown in Fig. 7.

The geometry accuracies of all rotational components of the linac have been assessed by performing Wintson–Lutz tests for gantry, collimator, and table rotations using RUBY phantom with Linac QA insert as well as the ball‐bearing phantom. Both systems were positioned according to the room lasers as well as using kV imaging. In addition, the RUBY phantom was positioned using CBCT imaging. It could be shown that the results obtained with the RUBY phantom and ball‐bearing phantom agree to each other. Furthermore, the results from this work demonstrated that with both systems, it is possible to check the coincidence between kV isocenter position and room lasers as well as kV isocenter position and MV isocenter position.

Image registrations between the MR and CT image datasets of the RUBY phantom with the System QA insert as well as of the STEEV Phantom with MRI insert did not yield clinical acceptable results in Masterplan using the mutual information algorithm with default settings. Since the registrations were successful in Monaco, this indicates distinctive implementations of image registration algorithms in different TPS and further evaluations should be carried out to identify the usability and the limit of the RUBY phantom as a QA device for TPS image registration of MR and CT datasets. Couch corrections computed based on CBCT image registration at the linac using RUBY phantom in combination with the System QA insert agree to the expected value within 1 mm and hence are comparable to the results demonstrated with the Linac QA insert. The same accuracy could be achieved using the STEEV phantom with the target insert, which is not surprising due to the high contrast details of the internal phantom structures.

Point dose measurements carried out for eight VMAT treatment plans, including two non‐coplanar radiations, are compared to calculated values in Masterplan using both the pencil beam and collapsed cone algorithms and Monaco using Monte Carlo engine. The mean absolute deviations of measurements for all radiation arcs performed using RUBY phantom with microDiamond detector and PinPoint 3D chamber as well as STEEV phantom with microDiamond detector are (1.1 +/− 0.6)%, (1.4 +/− 0.8)%, and (1.5 +/− 1.0)%, respectively, from Masterplan collapsed cone algorithm; (1.5 +/− 0.9)%, (1.7 +/− 1.0)%, and (2.1 +/− 1.3)%, respectively, from Masterplan pencil beam algorithm; and (1.0 +/− 0.8)%, (1.1 +/− 1.14)%, and (2.2 +/− 1.7)%, respectively, from Monaco Monte Carlo engine. Generally, larger deviations are observed between measurements and Masterplan pencil beam algorithm and the smallest deviations are observed between measurements and Monaco Monte Carlo engine. Comparable results have been shown for STEEV phantom with PinPoint 3D measurement compared to Eclipse with AAA algorithm in Villani et al.^8^ Nevertheless, the latter is not always the case for the STEEV phantom as shown in Fig. 6.

The good agreement within 2% between the microDiamond detector and PinPoint 3D chamber for all studied plans in this study is somewhat astonishing as both detectors have shown to exhibit different behaviors in small photon fields. The microDiamond detector has been shown to overrespond in small fields due to the presence high‐density detector components and the signal originated from charge imbalance in the conductive detector components.^9^, ^10^, ^11^ The air‐filled PinPoint 3D chamber underresponds in small fields due to the volume‐averaging effect and the low‐density sensitive volume.^12^, ^13^ Nevertheless, no correction factors have been applied to the measurements due to the lack of these factors for composite fields. Despite the small target volumes of plan 3 to 8 that correspond to the typical situations of stereotactic radiations, the consistency between the two detectors and the good agreement to TPS calculations indicate that both detectors are suitable for such point dose measurements within the scope of system QA. In these cases, the uncertainty due to the detector‐specific field size‐dependent dose response falls within the acceptance criteria of 3% of the system QA. Nevertheless, extra precautions must be taken when these tests are performed for treatment plans of target volumes with size comparable to the dimensions of the used detectors. Since system QA is not intended to substitute treatment plan verification, but more to test the system dependencies and to identify flaws within the radiation therapy process, independent 2D or 3D dose measurements using detector arrays are still recommended for individual plans to allow for more comprehensive comparisons between actual and calculated dose distributions.

## 5 Conclusion

A comprehensive evaluation of the RUBY modular phantom has been carried out as a routine linac QA phantom to check the integrity of the clinical IGRT workflow and the congruence of machine isocenter, imaging isocenter, and room lasers. Additionally, a system QA protocol has been derived using the RUBY phantom to check the IGRT‐based VMAT and stereotactic radiations workflow at an Elekta Synergy linac. The linac QA results obtained with the RUBY phantom have been benchmarked against widely used standard devices such as the Penta‐Guide phantom and the ball‐bearing phantom. The point dose measurements performed with the RUBY system QA insert show agreement better than 3% for most cases with the expected values from the TPS calculations. The modular construction of the RUBY that integrates the possibility to perform different routine QA in a single phantom is advantageous as it allows a more synchronous and harmonic QA workflow within a department regarding imaging protocol, positioning procedure, and image evaluation.

## Conflict of Interest

Daniela Poppinga is an employee of PTW Freiburg, Germany.

## References

[1] Klein EE , Hanley J , Bayouth J et al Task group 142 report: quality assurance of medical acceleratorsa. Med Phys. 2009;36:4197–4212. 19810494 10.1118/1.3190392

[2] Bissonnette J‐P , Balter PA , Dong L et al TG‐179 quality assurance for image‐guided radiation therapy utilizing CT‐based technologies. Med Phys. 2012;39:1946–1963. 22482616 10.1118/1.3690466

[3] Smilowitz JB , Das IJ , Feygelman V et al AAPM Medical Physics Practice Guideline 5.a.: Commissioning and QA of Treatment Planning Dose Calculations — Megavoltage Photon and Electron Beams Medical Physics Practice Guideline. J Appl Clin Med Phys. 2015;16:14–34. 26699330 10.1120/jacmp.v16i5.5768 PMC5690154

[4] Miften M , Olch A , Mihailidis D et al Tolerance limits and methodologies for IMRT measurement‐based verification QA: recommendations of AAPM Task Group No. 218. Med Phys. 2018;45:e53–e83. 29443390 10.1002/mp.12810

[5] Lutz W , Winston KR , Maleki N . A system for stereotactic radiosurgery with a linear accelerator. Int J Radiat Oncol • Biol • Phys. 1988;14:373–381. 3276655 10.1016/0360-3016(88)90446-4

[6] Dimitriadis A , Palmer AL , Thomas RAS , Nisbet A , Clark CH . Adaptation and validation of a commercial head phantom for cranial radiosurgery dosimetry end‐to‐end audit. Br J Radiol. 2017;90:1–9. 10.1259/bjr.20170053 PMC5602186 28452563

[7] Sykes JR , Lindsay R , Dean CJ , Brettle DS , Magee DR , Thwaites DI . Measurement of cone beam CT coincidence with megavoltage isocentre and image sharpness using the QUASAR^TM^ Penta‐Guide phantom. Phys Med Biol. 2008;53:5275–5293. 18758000 10.1088/0031-9155/53/19/002

[8] Villani D , Mancini A , Haddad CMK , Campos LL . Application of optically stimulated luminescence ‘nanoDot’ dosimeters for dose verification of VMAT treatment planning using an anthropomorphic stereotactic end‐to‐end verification phantom. Radiat Meas. 2017;106:321–325.

[9] Looe HK , Poppinga D , Kranzer R et al The role of radiation‐induced charge imbalance on the dose‐response of a commercial synthetic diamond detector in small fi eld dosimetry. Med Phys. 2019;46:2752–2759. 30972756 10.1002/mp.13542 PMC6849526

[10] Bouchard H , Seuntjens J , Duane S , Kamio Y , Palmans H . Detector dose response in megavoltage small photon beams. I. Theoretical concepts. Med Phys. 2015;42:6033–6047. 26429279 10.1118/1.4930053

[11] Looe HK , Harder D , Poppe B . Understanding the lateral dose response functions of high‐resolution photon detectors by reverse Monte Carlo and deconvolution analysis. Phys Med Biol. 2015;60:6585–6607. 26267311 10.1088/0031-9155/60/16/6585

[12] Poppinga D , Delfs B , Meyners J , Harder D , Poppe B , Looe HK . The output factor correction as function of the photon beam field size ‐ direct measurement and calculation from the lateral dose response functions of gas‐filled and solid detectors. Z Med Phys. 2018;28:224. 28869164 10.1016/j.zemedi.2017.07.006

[13] Looe HK , Büsing I , Tekin T et al The polarity effect of compact ionization chambers used for small field dosimetry. Med Phys. 2018;45:5608–5621. 30294821 10.1002/mp.13227

